# Far from Equilibrium Percolation, Stochastic and Shape Resonances in the Physics of Life

**DOI:** 10.3390/ijms12106810

**Published:** 2011-10-14

**Authors:** Nicola Poccia, Alessio Ansuini, Antonio Bianconi

**Affiliations:** 1 Department of Physics, Sapienza University of Rome, P. le A. Moro 2, 00185 Roma, Italy; E-Mails: nicola.poccia@roma1.infn.it; 2 International School for Advanced Studies, Trieste, Via Bonomea 265, Italy; E-Mail: ansuini@sissa.it

**Keywords:** emergence of life, percolation, complexity, non-equilibrium thermodynamics, criticality, shape resonance, Fano resonance, Feshbach resonance, stochastic resonance, quantum entanglement

## Abstract

Key physical concepts, relevant for the cross-fertilization between condensed matter physics and the physics of life seen as a collective phenomenon in a system out-of-equilibrium, are discussed. The onset of life can be driven by: (a) the critical fluctuations at the *protonic percolation* threshold in membrane transport; (b) the *stochastic resonance* in biological systems, a mechanism that can exploit external and self-generated noise in order to gain efficiency in signal processing; and (c) the *shape resonance* (or Fano resonance or Feshbach resonance) in the association and dissociation processes of bio-molecules (a quantum mechanism that could play a key role to establish a macroscopic quantum coherence in the cell).

## 1. Introduction

Today we know nearly all molecular components of a minimal cell and their interactions using novel microscopy and spectroscopy methods taking advantage of synchrotron radiation, lasers, magnetic resonance using photons from x-ray to radiofrequency, neutrons and quantum effects such as in atomic force microscopy and the Förster resonance energy transfer (FRET). The extraordinary discoveries of molecular biology have elucidated the structure of many organism genomes, revealing complex systems made of thousands of interacting genes. We have reached a fantastic knowledge on the complexity of the living cell thanks to advanced computers for storage and data analysis unveiling the networks of thousands of different bio-molecules in the cells. The collection of all these data, however, does not give us substantial clues on the overall cell functioning; therefore a number of scientists have started to think that it is becoming feasible to search for new physical laws of the living matter for the foundations of a new physics of life.

Founding the physics of the living cell means to find the general laws governing the cooperative network of networks of thousands of different selected biomolecules spanning a large time scale from picoseconds to years and space scales from nanometers to meters. The genomic data allows us to look for new ideas of evolution, which can be considered as a collective phenomenon far from equilibrium [1]. The global behavior depends on cooperative phenomena that cannot be explained at the microscopic level but are an emerging property of the system. New ideas and concepts need to be developed. The aim of this review paper is to focus on three known concepts of statistical physics and quantum mechanics that could play a key role in the foundations of a physics of living matter.

### 1.1. Percolation: The Careri Papers

Percolation is a critical behavior proposed by Giorgio Careri in [2] to be of relevance in order to understand the transition from the non-living phase to the living phase of the cell [3]. “Percolation” derives from a Latin word that means to filter or trickle through some medium, and commonly refers to the physical situation in which a fluid diffuses through a porous substance. Percolation phenomena are widespread in physics, chemistry, biology, earth sciences and also in environmental processes like forest fires [4–6]. During the last decades percolation has received great attention from mathematical research, for its probabilistic and analytic appeal [7], and also from interdisciplinary research, especially boosted by the discoveries in the field of complex networks and the processes (in particular diffusion) that can occur on interweaved topologies [8].

From the point of view of a theoretical physicist, percolation is interesting mainly as a model of critical behavior, but here we want to stress its relevance in biological processes. In 1986 G. Careri and collaborators [9] analyzed in several samples of lysozyme powders, the dependency of the electrical capacitance on the hydration, *i.e.* the concentration of water molecules in the samples. Capacitance depends on the properties of the material as a conductor of electricity that is carried by protons in this case. Careri *et al.* suggested that the protonic conductivity is a percolation process: protons move along threads of hydrogen-bonded water molecules that form a network spanning the whole system. This picture was suggested by the presence of a critical value of hydration—called percolation threshold *h_c_* —separating two regions: one with very low and constant capacity and the other characterized by a rapidly growing capacity, respectively below and beyond the threshold. The numerical value of *h_c_* was remarkably stable with respect to variations of the chemical properties of the system, like the pH or the chemical nature of the solvent. They interpreted these results as a signature of critical behavior: the dramatic change in the electrical properties was due to the sudden appearance of long-range correlations in the form of a macroscopic network (*i.e.*, ranging over the size of the whole sample) of microscopic conducting elements. The quantitative analysis of this threshold showed a percolation of protons along threads of hydrogen-bonded water molecules on the surface of lysozyme proteins.

In a subsequent paper, in 1989, Rupley *et al.* [10] proposed that the same theoretical picture could give an adequate explanation to the electrical features (capacitance and conductivity) of the cell membrane of *Halobacterium halobium*, an halophylic microorganism. The general picture emerging from their analysis was that ion transport in membranes can be based on pathways of randomly conducting elements, and a fixed simple geometry like a proton wire is not the only possible basis for a conduction mechanism. There is a cooperative transition at the critical hydration level for proton movement, and the behavior of the conductivity as a function of hydration in the neighborhood of the critical point is typical of a two-dimensional percolation process. In a related work the dependency of the dc conductivity on hydration was inspected for maize seeds [11], and an analogue mechanism of protonic conduction was found, reflecting the presence of a percolation network of water molecules through the biological matter at the onset of the living functions. These authors noted that the emergence of the connected network at the percolation threshold was in close correspondence to the onset of enzymatic functions and metabolic activity.

In conclusion, there may be a statistical component in the theoretical explanation of fundamental biological processes such as proton and ion transport across membranes: topological disorder and a statistical process may be needed in order to construct models with fundamental biological functions.

A major lesson from the statistical physics of critical phenomena is that the details of the structural chemistry of the system are not all equally important for the onset of collective phenomena [12]. The robustness and the universal features of percolation mechanisms rely on the statistical nature of the network formation process. In the case of protonic conduction, a collectivity of stochastically arranged conducting elements (which could be water molecules, but generally more any groups bearing labile hydrogen ions) self-organize into a network spanning the whole system [9–11]. Moreover these works imply a key role of lipid membranes in reducing the space dimensionality for the criticality of percolation in the cell from three dimensional (3D) to two dimensional (2D) space that could be relevant in pre-biotic evolution of living matter.

### 1.2. Criticality

In recent years, research on biological and social systems has seen the unprecedented availability of experimental data on the simultaneous state of a large number of interacting units, like for example the electrical recordings of neuronal activity from large retinal patches [13], the expression level of thousands of genes in microarrays [14], the position and velocity of hundreds of flocking starlings making complex and fascinating patterns in the sky [15], or the online collective activity of Internet users, exchanging opinions and ideas [16].

In [17] it has been suggested that in many of these cases the theoretical models emerging from the data are poised at a very special working point in the parameter space: the critical point. Maybe a deep theoretical principle is behind such different findings, spanning over many biological scales, from the single protein level to the whole organism and beyond, to societies. After all, in order to survive biological systems must process external information in an efficient and reliable way. When a system is at a critical point it is particularly sensitive to fluctuations of the external fields. In a critical state, these perturbations can propagate through the whole system: this is a premise for efficient information processing of the environmental changes by the system.

## 2. Percolation: Mathematical Description

In this section we present briefly what percolation theory is about and its main concepts. We refer to the excellent book of Stauffer [6] for a complete and enjoyable exposition of the theory.

**Figure 1:**
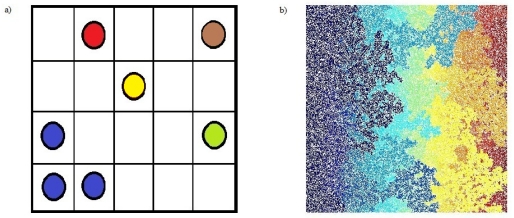
(**a**) Definition of percolation and clusters. Some squares are occupied by big dots and some are empty. The clusters are groups of neighboring occupied sites; we filled the dots inside a cluster with the same color. (**b**) Matlab simulation of two-dimensional (site) percolation at the percolation threshold (*P*_c_ = 0.5927) on a square lattice of size *L* = 10^3^. There is a cluster (the dark blue one) spanning the whole system from the bottom to the top of the square.

Let us imagine a large square lattice. Each square site can be in two mutually exclusive states: occupied by a big dot or empty (see Figure 1a). This is a good abstraction for many physical situations: for example, we can imagine that an occupied site is filled with a conducting material like copper and an empty site is filled with an insulator like rubber. A *cluster* is a group of neighbor squares occupied by these big dots (two squares are neighbors if they share an edge in common, if they just touch at a corner they are not neighbors and are sometimes called next-nearest neighbors). All couples of sites within a cluster are connected by an unbroken chain of neighbors. Percolation theory deals with the number and the geometrical properties (dimension, shape) of these clusters. The simplest percolation model is obtained when each site is occupied with a certain fixed probability *p* independently from the state of occupancy of all the other sites. It is clear that when *p* is equal to zero there are no clusters, because there is no site occupied, and when *p* is small the probability that a large cluster is created is very low. Raising *p* the clusters tend to increase their sizes and when a peculiar value, called “percolation threshold” *P_c_* is approached, a cluster whose dimensions are comparable to the system size appears.

Let us call *m_s_* the probability that a site is part of a cluster of size *s*. This probability gives us a great deal of information about the structure of the system. It can be easily guessed, from inspection of limiting cases (like for example the one-dimensional case in which the lattice reduces to a linear chain), that for large *s* this probability is dominated by an exponential decay *m_s_* ∝ *e*^−^ *^cs^* where *c* is a function of *p*. In order to proceed further and to find the correct functions that describe the dependency of *m_s_* on *p* an assumption that takes the name of *scaling hypothesis* has to be made:

(1) $$m_{s}=s^{-\tau +1}f\left[(p-p_{c})s^{\sigma }\right]$$

The functional form of *f* can be explicitly calculated only in special cases, and in the general case (like for two and three dimensional systems) has to be determined numerically. Two exceptions are represented by the linear chain, in which one finds *f*(*z*) = *e^z^* *f* (*z*)= *e^z^* (with τ = 2, σ = 1 ) and the infinite-dimensional case (realized on a particular topology called the Bethe lattice).

From Equation 1 and the experimental knowledge of *f* all the macroscopic information about the system in the neighborhood of the critical point can be obtained. Let us now turn back to the example of a conducting material. As a consequence of the scaling hypothesis the conductivity must be a power of the distance from the percolation threshold. In [10] (Equation 2), the following scaling assumption is made for the dc conductivity:

(2) $$k(p-p_{c})={\left(p-p_{c}\right)}^{t}$$

where *t* is the critical exponent of the transition, fitting with an excellent agreement the experimental data and suggesting that the protonic conduction is a percolation process where protons find their way along the sample through the percolating cluster. The quantitative analysis is based on the results on the conductivity of mixtures of conducting and non-conducting spheres in two and three dimensional systems and the critical exponent *t* is very similar to that of a surface (two dimensional) percolation.

An important lesson from percolation is that near the transition most quantities obey scaling laws that are almost universal in that they do not depend on the details of the lattice structure (be it triangular or squared) and other microscopic parameters. The dimensionality of the problem, on the contrary is a truly relevant parameter. This is a common feature of phase transitions and its explanation requires sophisticated techniques of renormalization group analysis that we cannot address here, but we observe that universality in critical phenomena can be clearly addressed mathematically and conceptually within the framework of limit theorems of probability theory.

We will see in the next section that randomness is a crucial component also in other biological processes where stochastic fluctuations are exploited in order to gain efficiency in information processing.

## 3. Stochastic Resonance

### 3.1. Biological Systems and the Benefits of Noise

Noise in human-made devices has been considered for long time as something to avoid or to reduce as much as possible. In electronic devices for example noise can affect the reliable communication between the electrical components and it can be a source of failures. In biological systems the situation can be profoundly different. In this context the noise can have different origins. First of all living organisms operates at non-zero temperature, and indeed thermal noise is present everywhere in cellular processes like the diffusion of proteins in chemoreception [18] or the electrical currents in ionic channels [19]. A second origin of noise is exemplified by the spontaneous activity of neurons in the brain. Here the excitation of a postsynaptic neuron membrane can be elicited by large fluctuations in the global activity of thousands of presynaptic neurons: this is a truly collective phenomenon [20].

Ranging from the whole organism to single cells and their microscopic constituents, biological systems exploit the noise as a resource with different astonishing mechanisms. Important examples are molecular motors, *i.e.*, motor proteins that can convert directly the chemical energy furnished by ATP consumption into mechanical work [21]. In doing so, they rectify the thermal noise, producing a directed motion that plays a key role in cellular transport. Another way in which biological systems can exploit the benefits of noise is through the mechanism of stochastic resonance, which operates mainly as a signal amplifier powered by noise, exploiting the nonlinearities of the system [22]. The functioning of molecular motors and the mechanism of stochastic resonance can be understood using the tools of statistical physics and of the theory of stochastic processes. In what follows, we will discuss briefly how stochastic resonance works.

### 3.2. Brief Description of the Mathematical Model

**Figure 2:**
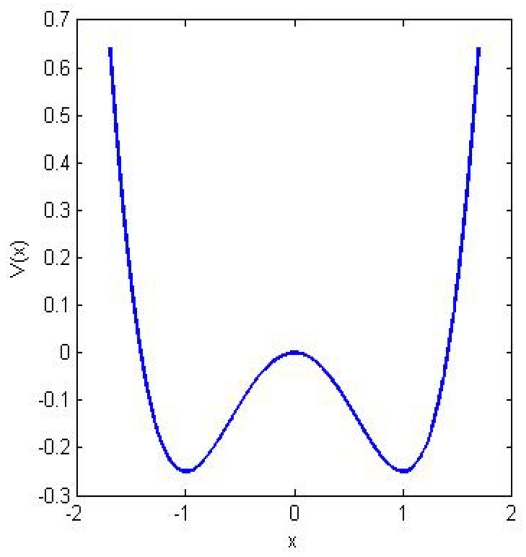
The mechanism of stochastic resonance. The double well potential *V*_(x)_ represents a physical system with two stable states. In absence of noise a particle, which at one time is within one of the two wells, relaxes towards the intrawell minimum and then stops. When stochastic perturbation is added to this system the particle can hop between the two wells with a rate predicted by reaction-rate theory. If a weak periodic forcing is present the overall potential minimum changes periodically from one side to the other. Stochastic resonance happens when the typical escape time from the lower barrier and the periodic forcing are synchronized.

Let us consider a heavily damped particle of mass *m* immersed in a fluid with viscous friction γ and subject to a force generated by a symmetric double-well potential *V* like the one shown in Figure 2.

The particle is subject to a stochastic force that is due to the collisions with the molecules of the fluid at temperature *T*. The equation of motion in the overdamped limit becomes

(3) $$m\ddot{x}=-\gamma \dot{x}+\sqrt{ɛ}\xi_{t}$$

where ζ_t_ is a normalized white noise (*i.e.*, with zero mean and unitary standard deviation) and ɛ is a parameter setting the amplitude of the fluctuational force. In the case of a particle immersed in a fluid we have ɛ = 2*D* where *D* is the temperature-dependent diffusion coefficient. The fluctuational forces cause interwell transitions with a rate given by Kramers reaction-rate theory [23]:

(4) $$r={ce}^{-\frac{\Delta V}{D}}$$

where Δ*V* is the height of the potential barrier separating the two minima. If we now apply to the particle a weak periodic forcing of the form *A*(*t*) = *A*_0_cos(Ω*t*) the double-well symmetric potential is tilted asymmetrically up and down, and in each instant of time the potential has two wells but with a different height. The hopping between the two periodically changing wells can then be synchronized with the weak periodic forcing. This synchronization takes place when the noise level is tuned at the value in which the typical waiting time between the transitions is comparable with half the period of the periodic forcing.

An asymptotic approximate formula (which is valid for long times and small amplitudes) for the system response is

**Figure 3:**
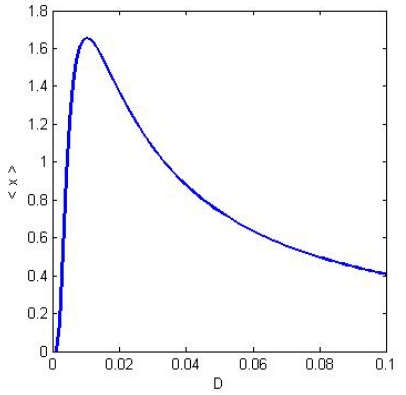
The typical shape of a stochastic resonance peak. The behavior of the amplitude of the system response <x> is plotted as a function of the noise level given by the diffusion coefficient *D*.

(5) E[x(t)]=〈x〉cos(Ωt-〈ϕ〉)

where *E* stands for the expectation value over the possible realizations of the stochastic noise. We see from (5) that the response of the system is sinusoidal with amplitude <x> and phase shift <ϕ>, and both these quantities are dependent on the diffusion coefficient or in other words the noise level. The amplitude <x> as function of the noise level shows clearly the stochastic resonance effect (see Figure 3). The concept of stochastic resonance has been introduced in the seminal paper by Benzi *et al.* [24], in order to explain the phenomenon of periodically recurrent ice ages. Since then stochastic resonance has been found in many physical systems, ranging from bistable ring lasers [25] to supercooled liquid water [26]. In biological systems stochastic resonance phenomena have been discovered at different spatial scales: in voltage-dependent alamethicin ionic channels [27] in ionic channels assemblies [28], in the mechanoreceptor hair cells of the crayfish *Procambarus Clarkii* [29,30] and in mammalian neuronal networks [31].

Different mechanisms have been proposed but a common feature emerges: the system experiences an increased sensitivity to small perturbations when the noise level is tuned to an optimal value [22,25-32].

### 3.3. Stochastic Resonance on Network Structures and in Biological Systems

In spatially extended systems, in which units interact through a certain interaction network, the noise is not the only thing that has to be tuned in order to have an optimal resonance: also the topology of the network is important. The optimal topology is a compromise between a scale-invariant network that enhances long-range connections and the presence of hubs, and a short-range connection topology [33]. Remarkably this is the case for the neuronal networks in mammalian neo-cortex, which is the biological substrate for the higher cognitive functions like memory and, in the case of our species, language. One of the fundamental organizing principles in the architecture of the associative cortex is the coexistence of two systems of connections between pyramidal neurons that contribute the largest part of the wiring material. Pyramidal neurons in the cortex receive input information by means of two dendritic systems (called basal and apical dendrites), conveying information respectively from neighboring (within hundreds of micrometers) and distant (several centimeters) neurons [34].

Stochastic resonance is a well-accepted paradigm in biological sciences, especially in neurobiology where information processing is the essential function. If we consider the case of the crayfish [30], its mechanoreceptor cells are specialized to detect weak movements of the water, allowing for the animal to detect its predators. This was the natural system in which stochastic resonance was observed for the first time; the following discoveries of analogue mechanisms in a wealth of other biological systems suggest the intriguing possibility that evolution has found a way to exploit the benefits of noise regulating the level of endogenous noise in an optimal way. In [29] it has been proposed that stochastic resonance can be a common phenomenon in sensory biology in which the sensors operates in presence of external or endogenous (as in the case of neurons) noise.

## 4. Shape Resonance

The living state of the cell is determined by the coherence in time and space of the biochemical reactions as proposed by G. Careri in the eighties [9]. The key point for the transition from the non living to living state of biological matter is the dynamical order of the biochemical events in the cell. The flexible proteins fluctuate in a complex landscape of multiple protein conformations performing association and dissociation molecular processes, aminoacids get associated in proteins and proteins dissociate in aminoacids and so on. The key point of the living state of the cell is how the specific biochemical molecules selected by the evolution are able to sustain this coherent multiscale and multi-phase living state at room temperature. In fact it is possible that the dynamical order in the biological networks [35] that manifest itself in evolution with quantum statistics features [36–38] could be driven by Feshbach resonances [39]. A large interest is growing recently on the quantum physics of living matter [40]. Quantum coherence is emerging in photosynthesis [41–45] in genetics [46–49] and avian magnetoreception [50,51], quantum darwinism [52] and the entanglement production in non-equilibrium thermodynamics [53] have been proposed. The biological networks show a large heterogeneity that reduces the network entropy [54] and quantum statistics has been found to be in action [55].

It has been proposed that a specific feature of the quantum world, the “shape resonance” called also “Fano resonance” or “Feshbach resonance” (since it was introduced by Fano and developed by Feshbach) could play a key role in the transition from the non-living matter to the dynamical order in the living matter [39,56] in the pre-biotic evolution.

### 4.1. A Brief History of the Shape Resonance

**Figure 4:**
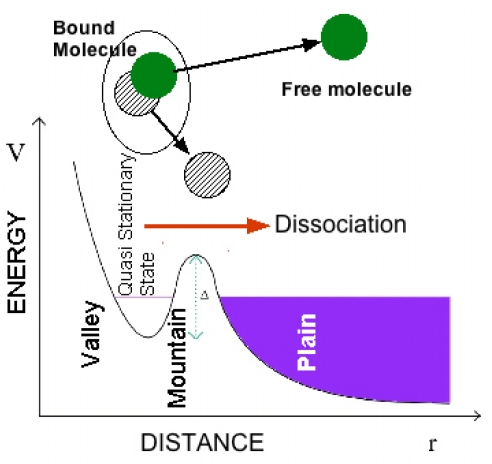
The dissociation process of a quasi-bound molecule in an excited predissociation state (called a quasi stationary state) in a potential valley degenerate with continuum states of the dissociated particles in the plain region of the potential as described by Rice [58,59].

Here we describe the evolution of the scientific idea of shape resonance to understand its relevance. At the time of the foundations of quantum mechanics in 1929 O. K. Rice in California noticed that “when the discrete vibration rotation absorption bands connected with transitions to a certain final electronic state of a molecule overlap the continuous region for the transitions to another final electronic state, some of the discrete bands may be diffuse, *i.e*., the rotation lines may be broad and blur into each other. The broadness of the lines has previously been assumed to be connected with the short life period of a molecule in a discrete state, when there is the possibility of its making a radiationless transition to a state of dissociation” [57]. He started to use the new formalism of quantum mechanics to explain atomic and molecular collisions and the dissociation of a diatomic molecule induced from a molecular rotational excited state. The molecular dissociation is considered as a manifestation of tunneling from a potential “valley” through a “mountain” into the “plain” (see Figure 4) in his papers [58] written during a visit in the Heisenberg’s institute in Leipzig and he discussed also the analogy with the nuclear alpha decay [59,60].

**Figure 5:**
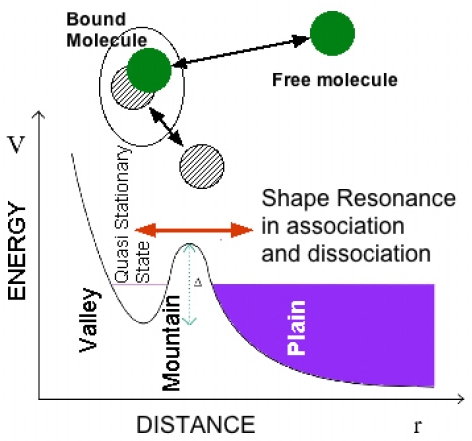
The “shape resonance” is determined by the quantum configuration interaction between the quasi-bound states forming the bound molecules in the potential *valley* and the dissociated free molecules moving in the regions of the potential *plain*. The shape resonance modifies the wave function of the free particles and it controls the interaction between the free molecules.

Around the same time also Ettore Majorana developed the theoretical idea of G. Wentzel for the radiationless quantum jumps in the Auger decay introducing the idea of configuration interaction between a discrete and a continuum set of states. In this new quantum scenario the quasi-stationary bound states interfere with the continuum states as shown in Figure 5. Majorana has used these ideas in one paper on the atomic auto-ionization processes *i.e*., the two electrons photo-absorption lines above the ionization potential in atomic spectra [61]. Majorana was also attracted by the nuclear physics problem of α decay: the artificial disintegration of nuclei by bombardment with alpha-particles as a quantum phenomenon, but he did not publish his works [62–64]. Fano in 1935 developed the theory of the “shape resonance” due to interference effects between a closed and an open scattering channel for the two electron excitations above the ionization potential in Helium [65,66]. He has been able to derive the formula for the anisotropy of the line-shape of atomic absorption cross-section. The anisotropy parameter was shown to be a good measure of the quantum interference between the bound and the continuum states. Later the idea of “shape resonance” was developed by Feshbach for the configuration interaction between a large number of resonances in the scattering processes in nuclear physics [67]. With the advent of synchrotron radiation research the shape resonances became an active research field in the physics of excited localized states degenerate with the continuum in the near edge x-ray absorption structure (XANES) of molecules, proteins and condensed matter [68–70]. Recent progress in modern nanotechnology allows one to scale down to nanoscale various important devices and Fano resonances have been recognized as a fundamental concept for the description of quantum transport in electronic processes in nanoscale structures [71].

In the eighties the association and dissociation problems found renewed interest in atomic collisions with a focus on molecular reactions like *H* +*H* _2_ →*H* _3_ →*H* +*H* +*H* that shows the key quantum mechanical feature of the configuration interaction between a bound state and a continuum described by the shape resonance (see Figure 5). The quantum interferences lead to exciting situations where long-lived metastable states (quasi-stationary states) coexist with ultrafast processes observed experimentally. This phenomenon is usually called the “trapping effect” or “avoided resonance overlapping” and has been studied in numerous fields of physics and chemistry [72–74] driven by the dynamics of a state resulting from the superposition of a discrete state with a continuum one.

The interference at the resonances occurs in the dissociation of van der Waals complexes and is particularly important in order to understand the fragmentation where overlapping resonances have been observed. The physical possibility of such a state is what could allow proteins to synchronize their interaction after a time t accordingly to the equilibrium constant describing the chemistry of the reaction. It is worth to mention that such behavior is only possible in a quantum mechanical formulation of the association-dissociation process among the proteins interactions.

Processes of association and dissociation have been later discovered in ultracold gases designing different experiments depending on the diatomic energy spectra. The ultracold molecules can be dissociated by ramping the magnetic field in the opposite direction through the Feshbach resonance. Once above the Feshbach resonance, the molecules dissociate spontaneously with a finite rate. The association of ultracold molecules with a Feshbach resonance can be used to obtain full quantum control of this chemical reaction [75,76]. Recently, studies have moved in search of Feshbach resonance dimers more closely linked and of Efimov resonance trimers. Observation is closely related to an atom-dimer resonance as predicted by Efimov [77]. Trimer state manifests itself in a resonant enhancement of inelastic collisions in a mixture of atoms and dimers, close to a Feshbach resonance [77–80], giving the opportunity to explore a new few-body physics.

Recently the shape resonance has been found in multigap superconductors [81–87] made of multiple condensates characterized by different order parameters (superconducting gaps). In these systems a first set of fermionic particles, with high density and weak interactions, forms a first large Fermi surface and a second set of particles, in a regime of low density and/or strong interactions, forms bosonic pairs in a second small Fermi surface pocket. The superconducting shape resonance in the gaps controls the exchange of bosonic pairs with BCS pairs. The shape resonance in the superconducting gaps appears in systems showing multiphase complexity [88] that has delayed the understanding of the mechanism of high *T*_c_. The complexity and shape resonances appear to be the key for keeping quantum coherence at high temperature. The time evolution of this complexity is now of high interest since its control will allow the development of advanced material with novel functionalities [89]. The complex structural multiscale phase separation of these systems appears similar to structural features of living matter [90].

### 4.2. A Brief Description of the Shape Resonance through Simple Examples

The quantum theory of configuration interactions between closed and open channels was applied to explain the lineshape of absorption spectra above the ionization limit of atomic spectra due to two electron excitations in He: *He*(1*s*^2^)+*hν* →*He*(1*s*^0^ 2*s*^1^*np*^1^ ) with mixing between quasi boud stationary state with continuum states *He*(1s^0^ 2*s*^1^*np*^1^ )↔*He*(1*s*^1^ )+*ɛ*p, where ɛ*p* is a free photoelectron, by Ugo Fano [65,66] who obtained the formula for the spectral line shape of the scattering cross-section:

(6) $$F(ɛ)=a\frac{{\left(q+ɛ\right)}^{2}}{{ɛ}^{2}+1}+b$$

**Figure 6:**
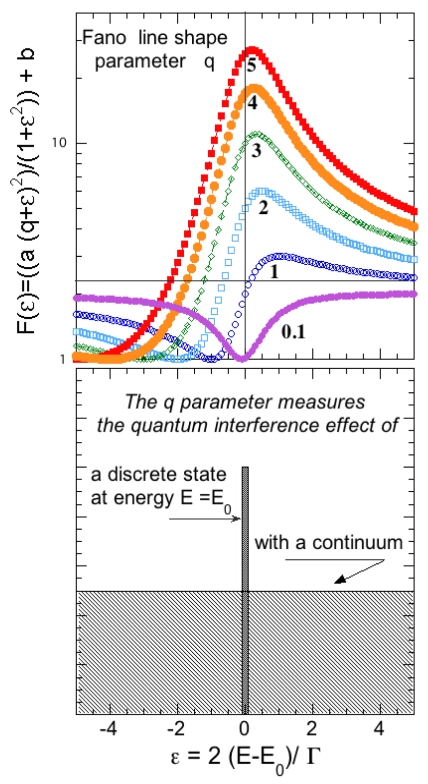
The evolution of the Fano line-shape for the shape resonance, as a function of the strength of the interference term proportional to 1*/q*, where *q* is the parameter of the Fano formula.

Using a phenomenological parameter *q* and a reduced energy ɛ defined by 2(*E* – *E*_0_)/Γ where *E*_0_ is the resonant energy and Γ is the width of the autoionized state, this equation predicts a maximum and a minimum in the Fano line-shape. The Fano formula is a superposition of the Lorentzian line shape of the discrete level $\frac{q^{2}-1}{{ɛ}^{2}+1}$ with a flat continuous background and a mixing term $q\frac{2ɛ}{{ɛ}^{2}+1}$ The formula 6 describes well the experimental atomic photoabsorption cross-section for the excitations of two electrons above the continuum threshold or the cross-section for the capture of a free neutron captured into the nucleus around the energy of the nuclear shape resonance. The deviation of the cross-section from the Lorentzian is measured by the Fano-line-shape parameter *q*. The value of *q* can be easily extracted form experiments. For a very large value of *q* the line shape approaches a Lorentzian because of the lack of quantum interference between the states in the continuum and the quasi-bound states as shown in Figure 6. By decreasing the value of *q* a minimum develops at energies lower than the energy of the quasi bound state at *E*_0_ and the maximum moves well above the quasi-bound state. In the extreme case of strong interference, proportional to 1*/q*, the minimum of the cross-section moves close to the quasi bound state energy *E*_0_ and becomes very large while the maximum moves far away from *E*_0_ and its intensity vanishes. The result is that for very high interference the shape resonance gives essentially an anti-resonance driven by the negative interference effects.

The shape resonance can be understood through a classical mechanical analogue. Let us consider a pair of classical oscillators ω_1_ and ω_2_ connected by a spring. If some energy is injected into one of the oscillators at *t =* 0 it will then flow into the second one, while the first will go back to the resting state. After a while the energy from the second oscillator will go back to the first oscillator and so on. The resonant exchange becomes larger as the frequency difference *ω*_1_ −*ω* _2_ → 0. In quantum physics the analogue case is the configuration interaction between two bound states confined in the same atom or in the nucleus and it depends on the energy difference between the levels.

**Figure 7:**
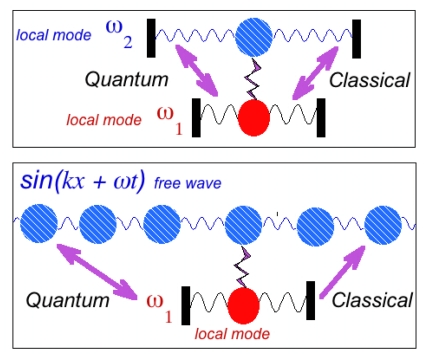
(Upper panel): A pair of oscillators ω_1_ and ω_2_ is weakly linked. The energy injected into one oscillator flows to the other one and *vice versa* both in the classical world and in the quantum world. (Lower panel): A chain of oscillators has a weak link with a local oscillator. The energy given to the local oscillator flows towards the chain of oscillators, moves away as a wave sin(*kx +* ω*t*) and does not come back to the local oscillator in classical physics. There is no resonance in classical physics between the localized mode and the free wave. On the contrary, in the quantum world the shape resonance describes the resonance between the free wave and the local mode.

There is not such a resonance in classical physics between the localized mode and the free wave. The lower panel of Figure 7 shows a chain of oscillators coupled to a local oscillator. If energy is injected into the local oscillator the energy flows into the chain of oscillators while the local oscillator goes back to the resting state. The energy given to chain of oscillators flows away as a wave, sin(*kx* + *ωt*) and it does not come back to the local oscillator.

On the contrary, according to Fano, the quantum world shows the shape resonance between the free wave and the localized mode. The difference between the energy of the free wave and the energy of the local oscillator is the tuning parameter.

A second classical case where the anisotropic line-shape appears is the case where an external periodic force of variable frequency ω is applied to the pairs of oscillators with different frequencies shown in the upper panel of Figure 7 (see ref. [91]). The equations of motion in the case of the forced oscillators are given by:

(7) $$\begin{array}{l} {\ddot{x}}_{1}+\gamma_{1}{\dot{x}}_{1}+\omega_{1}^{2}x_{1}+v_{12}x_{2}=a_{1}e^{i\omega t}\\ {\ddot{x}}_{2}+\gamma_{2}{\dot{x}}_{2}+\omega_{2}^{2}x_{2}+v_{12}x_{1}=0 \end{array}$$

where v_12_ describes the strength of the coupling between the two oscillators. Solving this set of equations and calculating the amplitudes, one finds that the resonant behavior of the amplitude as a function of the frequency of the external force has two peaks (symmetric and asymmetric line-shape) near the two “eigen” frequencies. The coupled oscillator responds with two resonances 1) a symmetric resonant profile at *ω*_1_ and an asymmetric profile at *ω*_2_. The first is described by a Lorentzian function (known as a Breit-Wigner resonance) and the second is characterized by the asymmetric Fano line-shape as an effect of the phase interference of the two oscillators when driving frequency passes through the resonance.

A solvable example of a resonance between a chain of oscillators and a local mode is the Fano-Anderson Hamiltonian:

(8) $$H=\sum_{n}C\varphi_{n}\varphi_{n-1}^{*}+E_{b}|\psi {|}^{2}+J\psi^{*}\varphi_{0}+c.c$$

This model describes the interaction of two subsystems [71]. One subsystem is a linear discrete chain with the complex field amplitude *φ_n_* at site *n* and nearest-neighbor coupling with strength C. This system supports propagation of plane waves with dispersion ω*_k_* = 2*C* cos *k*. The second subsystem consists of a single state *ψ* with energy *E*_b_. The third term the coupling among the chain and the oscillator given by the coupling coefficient *J* between the state *ψ* and the state *φ*_0_ of the discrete chain. A propagating wave may directly pass through the chain or instead visit the Fano state, return back, and continue with propagation. The main resulting action of the Fano state is that the strength of the effective scattering potential *J*^2^/(ω*_k_* − *E*_b_)resonantly depends on the frequency of the incoming wave ω*_k_*. If *E*_b_ lies inside the propagation band of the linear chain |*E*_b_| < 2*C*, the scattering potential will become infinitely large for ω*_b_* = *E*_b_, completely blocking propagation as shown by the strong anti-resonance with a dip in Fig. 6 at high strength of the mixing term. Therefore meeting the resonance condition, leads to a resonant suppression of the transmission, which is the main feature of the Fano resonance.

The asymmetric Fano lineshape of the shape resonance, where both the anti-resonant suppression and the resonant enhancement of the wave transmission are located close to each other is obtained by an extension of the basic Anderson model. In fact, each degree of freedom introduced in the single oscillator site provides an additional local path for the scattering wave to propagate, which may lead to a variety of interference phenomena.

This approach is said to derive from the Fano-Anderson Hamiltonian in solid-state physics introduced in 1961 to describe localized magnetic states in metals [92]

(9) $$H={ɛ}_{c}b^{+}b+\sum_{k}\left[{ɛ}_{k}c_{k}^{+}c_{k}+A_{k}\left(c_{k}^{+}b+b^{+}c_{k}\right)\right]$$

The Hamiltonian (9) is often used to describe heavy fermion systems and Kondo insulators [93] and is called “Anderson impurity model”. Following Mahan [94] it is called the “Anderson or the Fano model depending on whether the speaker is a solid state or atomic physicist”. The first term of (9) describes a localized state at fixed energy ɛ*_c_* and operators *b^+^* and *b*. This state is usually called impurity. The second term describes a continuous set of states with energy ɛ*_κ_* and *k* wave-vector and operators *c^+^* and *c* that have a finite band-width given by the tight-binding models of solids or by a free-electron model. The third term describes the mixing between these two kind of states describing the process where the continuum particle hops onto the impurity (*b*^+^*c_k_*) or particle hops off the impurity into the continuum (*c_k_* ^+^*b*) that describe the scattering resonance for the particle at the Fermi level. The particles can be bosons or fermions, in this last case they preserve the spin orientation jumping from the bound state to the continuum and vice-versa.

However the key feature of the “shape resonance” is the mixing of different many body wave-functions introduced by the quantum mechanics formalism of Fano and Feshbach.

The clearest configuration interaction between many body functions is the case of shape resonance in the superconducting gaps [84–87] arising from the mixing between a Bardeen-Cooper-Schriffer (BCS) condensate in a first band *i* with a Bose-like condensate in a different band *j* with a finite width near a Lifshitz transition [87]. The interference term, called the “interband pairing” transfers a pair of spin up and spin down particles from the first *i* to the second *j* condensate and vice-versa. The extension of the BCS theory to overlapping bands b and c in complex systems [95] is given by the Hamiltonian (10).

(10a) $$\begin{array}{l} H=\sum_{k}{ɛ}_{kc}c_{k}^{*}c_{k}+\sum_{k}{ɛ}_{kb}b_{k}^{*}b_{k}-\sum_{k,k^{\prime }}V_{cc}c_{k↑}^{*}c_{-k↓}^{*}c_{-k^{\prime }↑}c_{k^{\prime }↓}-\sum_{k,k^{\prime }}V_{bb}b_{k↑}^{*}b_{-k↓}^{*}b_{-k^{\prime }↑}b_{k^{\prime }↓}\\ -\left(\sum_{k,k^{\prime }}J_{cb}(k,k^{\prime })c_{k↑}^{*}c_{-k↓}^{*}b_{-k^{\prime }↑}b_{k^{\prime }↓}+\sum_{k,k^{\prime }}J_{bc}(k,k^{\prime })b_{k↑}^{*}b_{-k↓}^{*}c_{-k^{\prime }↑}c_{k^{\prime }↓}\right) \end{array}$$

The first two terms give the kinetic energy ɛ*_kb_* and ɛ*_kc_* of the two bands and *c*, c*, *b* and b* are the corresponding annihilation and creators operators. The third and fourth terms *V_cc_*, *V_bb_*, are the weak electron-electron attraction forming the cooper pairs and giving the BCS condensates in each band. The summations extend only over different *k⃗* values in the two bands corresponding to energies with a distance ± *Ħ ω* of the Fermi surface. The fifth and sixth terms are the Fano-like mixing terms due to mixing of the two condensates with the transfer of a pair from the b to c band and vice-versa.

Here each macroscopic condensate is characterized by an order parameter, the superconducting gap, that can be measured and the interband term acts as the link between the wave-functions of the two or more condensates giving a single critical temperature. J. Kondo in 1961 proposed [96] that the last mixing terms ∑*_k,k_*_′_ *J* (*k,k*′) (b^*^_k__↑_ b^*^ _−_*_k_*_↓_ *c*_−_*_k_* _′↓_*c_k_* _′↑_ +*c.c.*) where the exchange integral *J* (*k*, *k*′) may be repulsive or attractive is responsible for the increase of the transition temperature in complex multi-Fermi surfaces giving multigap superconductors [81,87] also in the case where pairs are formed only in one of the two band. In the extreme case of a first bosonic set of states (hard core bosons “*b*”) and itinerant electrons “*c*” the Hamiltonian is given by [97]

(10b) $$H=\sum_{k}({ɛ}_{ck}-\mu )c_{k}^{*}c_{k}+2\sum_{i}(E_{b}-\mu )b_{i}^{*}b_{i}-\sum_{i,j}V_{ij}b_{i}^{*}b_{j}+\left(\sum_{k,q}J_{q}(k)c_{k↑}^{*}c_{-k↓}^{*}b_{q}+h.c.\right).$$

where *c* * and *b* * are the creation operators of electrons in the Fermi surface and bosonic pairs, respectively. In this model of a narrow set of bosonic states degenerate with the Fermi level of the free particles a strong mixing depletes the quasi-particle spectral function of the free particles and induces a pseudogap in the large Fermi surface that does not favor the gap amplification. This pseudogap effect corresponds to the negative interference effect in the Fano-line-shape as shown in Figure 6. It has been recently shown [87] that the shape resonance giving an amplification of the critical temperature occurs in the case of a finite bandwidth of the second states *j*. It takes place for a finite shift of its band edge from the Fermi level of the free particles of the order of the inter-particle interaction. Therefore the positive effect on the critical temperature is controlled by a fine tuning of the chemical potential like in the Fano line-shape shown in Figure 6.

### 4.3. Shape Resonance in Biology

The concepts of shape resonance may play a role in biomolecules association and dissociation. Using the previous simple oscillator examples, we can develop some concrete examples in biology.

A zinc finger protein is a good candidate for creating a quasi-stationary state while it is transiently in contact with DNA. The oscillator chains, described in the previous section, would be the idealized case of the zinc finger protein leaving far away in the cell from DNA, while the peculiar state where the zinc finger protein is in contact with DNA would represent the single oscillator coupled to the chain. The more are the degrees of freedom linked to this state such as transcription factors and other biomolecules, the more would be the complex interference pattern possible. Instead, if no shape resonating mechanism is working, the zinc finger would leave DNA without interacting with it anymore, since the energy flow would be dissipated off immediately after the reaction. On the other hand, if the shape resonance mechanism is possible, we could expect the zinc finger proteins behaving as a wave leaving to the infinite, and returning after a finely tuned time back to the DNA in a synchronized fashion.

In metabolic networks nodes are small molecules (substrates) and links are chemical reactions controlled by specific enzymes [35]. This representation reveals that protein-protein interaction networks are not randomly organized and display properties that are typical of hierarchical networks, combining modularity and local clustering to scale-free topology.

It is known that in protein-protein networks, two proteins may interact and form a new complex depending on whether they are linked by a link. Links in networks therefore suggest a transient presence of an associated state between two proteins. If a node A has more than a link, for example two, this means that the same protein (node A) can associate and dissociate at subsequent times with two different proteins B and C. Each link is weighted [98] by the proper equilibrium reaction constant which describes quantitatively the rate of association and dissociation.

(11) B+A+C→B+AC→BA+C→A+B+C

In this simple example therefore we have two different quasi-bound states AC and BA for the same protein A and each of these states is characterized by an association-dissociation equilibrium constant.

It is an empirical fact that a protein after some time leaves the protein with which it has interacted. It is therefore possible to imagine the leaving protein as a wave that scatters at infinity. In other words, the protein spends some time near the protein to which is linked, but eventually ends up far from it.

The links attached to the node can belong to the same network or be in a modular region between overlapping networks, each of them performing a variety of tasks. However, when a first node protein is transiently associated with a second linked protein, the third protein becomes dissociated and free in solution.

However, how can we try to explain the dynamical process which forbids the free proteins to bind with the many other highly concentrated proteins in a typical cell milieu? We propose that the protein-protein interaction has to be tuned (changing physical parameters such as density, temperature, membrane architectures and pH) near a shape resonating state. In this resonating state it is not essential how far the protein is when it is dissociated because it will return to associate transiently again.

A condensation of a biochemical reactions network develops if the same protein associate and dissociate transiently in time with a great number of proteins. In other word, this means that the protein is a highly linked node of the network. The link condensation can be controlled by weighting the links [98] themselves with the appropriate association and dissociation constants. Association and dissociation of protein in the network is a common process and it is the starting point in order to understand the dynamics of biological networks.

In chemistry and biochemistry, a dissociation constant is a specific type of equilibrium constant that measures the propensity of a larger object to separate (dissociate) reversibly into smaller components, as when a complex falls apart into its component molecules, or when a salt splits up into its component ions. The dissociation constant is usually denoted *K_d_* and is the inverse of the association constant, that in the special case of salts can also be called an ionization constant. For a general reaction:

(12) $$A_{x}B_{x}\Leftrightarrow xA+yB$$

in which a complex *A_x_B_y_* breaks down into *x A* subunits and *y B* subunits, the dissociation constant is defined as

(13) $$K_{d}=\frac{{\left[A\right]}^{x}\times {\left[B\right]}^{y}}{\left[A_{x}B_{y}\right]}$$

where [*A*], [*B*], and [*A_x_B_y_*] are the concentrations of *A*, *B*, and the complex *A_x_B_y_*, respectively. At each time step new complexes can form and existing complexes can break down into smaller complexes according to experimental interaction rules provided as input to the model.

A careful engineering of the dissociation constants within network of protein interactions could be a way to achieve a shape resonance in biological matter, establishing finally a robust and functional system against the decoherence attacks of temperature [39].

Let us assume that there exists a metastable state in which the two proteins are linked for some time. After a certain amount of time the protein interaction terminates and the two proteins are again in free motion. In order to estimate the transition from the quasi-stationary state to the continuous state, we can describe the wave function of the two protein systems in the metastable state (quasi-stationary state)

(14) $$\psi_{QS}=\psi_{p1\_bound}\cdot \psi_{p2\_bound}$$

while in the continuum we have a many-body wave function of the following form:

(15) $$\psi_{mb}=\psi_{p1\_free}\cdot \psi_{p2\_free}$$

The transition amplitude is given by the exchange interaction (energy transfer: monopole)

(16) $$\int \psi_{QS}^{*}\cdot \psi_{mb}dr$$

that describes the oscillations between the quasi-stationary state and the free state of the proteins and in some energy range the shape resonances are expected to determine the coherence of the protein dynamics.

## 5. Conclusion

In this review, we have discussed three ingredients that could be of relevance in a physical theory of life. The first is the granular percolation of multiple phases far from equilibrium. Indeed, near a critical state, it has been shown that a fractal granular percolation plays a relevant role in the robustness of a coherent quantum state in high temperature superconductors [88]. The scenario of percolation was proposed by Careri *et al.* [9–11] in living matter and appears in the wavelike energy transfer through quantum coherence in complex photosynthetic systems. The second ingredient is stochastic resonance which takes advantage of the noise of the system: in the optimum range of noise, the granular system can undergo a stochastic resonance, lowering the overall entropy. Assisted energy transport in a noisy environment such as photosynthetic systems has been considered. Noisy environments are however characteristic also of fundamental biomolecules such as DNA. This is the framework where the third ingredient comes to aid: the shape resonance. The synchronization of the collectivity of biomolecules in the cell could be originated by finely tuned shape resonances. At a higher scale of biological organization, quantum entanglement has been proposed to optimize the orientation of flocks of birds [50,51] and stochastic-quantum treatment of Darwinian evolution is a subject of recent interest [99,100,36,37]. Finally the possibility that biological molecules have been selected by evolution because of their particular quasi-stationary states is discussed, invoking a possible role of the quantum shape resonances.
